# Resistance to glufosinate is proportional to phosphinothricin acetyltransferase expression and activity in LibertyLink^®^ and WideStrike^®^ cotton

**DOI:** 10.1007/s00425-015-2457-3

**Published:** 2016-01-05

**Authors:** Caio A. Carbonari, Débora O. Latorre, Giovanna L. G. C. Gomes, Edivaldo D. Velini, Daniel K. Owens, Zhiqiang Pan, Franck E. Dayan

**Affiliations:** Faculty of Agronomic Sciences, São Paulo State University, Botucatu, SP Brazil; USDA-ARS Natural Products Utilization Research Unit, University, MS 38677 USA; Colorado State University, Bioagricultural Sciences and Pest Management, Fort Collins, CO 80523 USA

**Keywords:** Ammonia, Glutamate, Photosynthesis, Marker gene, Pat, Bar, Injury, *Gossypium hirsutum* L, Glufosinate ammonium, Cotton

## Abstract

**Insertion of the gene encoding phosphinothricin acetyltransferase (PAT) has resulted in cotton plants resistant to the herbicide glufosinate. However, the lower expression and commensurate reduction in PAT activity is a key factor in the low level of injury observed in theWideStrike**^**®**^**cotton and relatively high level of resistance observed in LibertyLink**^**®**^**cotton.**

LibertyLink^®^ cotton cultivars are engineered for glufosinate resistance by overexpressing the *bar* gene that encodes phosphinothricin acetyltransferase (PAT), whereas the insect-resistant WideStrike^®^ cultivars were obtained using the similar *pat* gene as a selectable marker. The latter cultivars carry some level of resistance to glufosinate which enticed certain farmers to select this herbicide for weed control with WideStrike^®^ cotton. The potency of glufosinate on conventional FM 993, insect-resistant FM 975WS, and glufosinate-resistant IMACD 6001LL cotton cultivars was evaluated and contrasted to the relative levels of PAT expression and activity. Conventional cotton was sensitive to glufosinate. The single copy of the *pat* gene present in the insect-resistant cultivar resulted in very low RNA expression of the gene and undetectable PAT activity in in vitro assays. Nonetheless, the presence of this gene provided a good level of resistance to glufosinate in terms of visual injury and effect on photosynthetic electron transport. The injury is proportional to the amount of ammonia accumulation. The strong promoter associated with *bar* expression in the glufosinate-resistant cultivar led to high RNA expression levels and PAT activity which protected this cultivar from glufosinate injury. While the insect-resistant cultivar demonstrated a good level of resistance to glufosinate, its safety margin is lower than that of the glufosinate-resistant cultivar. Therefore, farmers should be extremely careful in using glufosinate on cultivars not expressly designed and commercialized as resistant to this herbicide.

## Introduction

The natural phytotoxin l-phosphinothricin is a bioproduct from the breakdown of bialaphos produced by *Streptomyces viridochromogenes* and *S. hygroscopicus* (Dayan et al. 2009; Dayan and Duke 2014). It is a non-selective herbicide that is applied post-emergence, with low translocation and a broad spectrum of weed control. Glufosinate, a synthetic mixture of the d- and l-form of phosphinothricin, is the only commercial herbicide that targets glutamine synthetase (GS), an enzyme directly related to nitrogen metabolism in plants.

l-Phosphinothricin, the active ingredient in glufosinate (the d-isomer has no biological activity), competes for the glutamate-binding site in GS, thus inhibiting the enzyme and leading to glutamine deficiency and highly toxic ammonia accumulation in plants (Dayan et al. 2015; Downs et al. 1994; Hess 2000; Lacuesta et al. 1990; Tachibana et al. 1986; Wild and Wendler 1991) as well as glutamate accumulation (Barberis 2012).

Inhibition of GS and accumulation of ammonia triggers a series of secondary effects, such as inhibition of the ribulose-1,5-bisphosphate carboxylase/oxygenase (rubisco) enzyme (Wild and Wendler 1993) and interference in the electron flow of the photosystem (Reddy et al. 2011), strongly affecting photosynthesis (Coetzer and Al-Khatib 2001; Wendler et al. 1990; Wild and Wendler 1991).

Glufosinate-resistant cotton cultivars have a *pat* or *bar* gene that codes for phosphinothricin acetyltransferase (PAT) enzyme production. The *pat* gene is very similar to the *bar* gene with an 87 % identity at the nucleotide sequence level and both encode PAT protein of 183 amino acids with 85 % amino acid sequence identity. Their molecular weights (approx 22 kDa) are comparable and they have similar substrate affinity and biochemical activity (Wehrmann et al. 1996). PAT detoxifies glufosinate ammonium by acetylation of the l-isomer into *N*-acetyl-l-glufosinate ammonium which does not inhibit GS (Dröge-Laser et al. 1994), thus inactivating it in plants (Hérouet et al. 2005; Tan et al. 2006).

LibertyLink^®^ cotton is resistant to glufosinate by overexpressing the *bar* gene derived from *S. hygroscopicus*, strain ATCC 21705, whereas the insect-resistant WideStrike^®^ cotton (expressing Cry1Ac and Cry1F genes) expresses the *pat* gene from *S. viridochromogenes* which confers some resistance to glufosinate (Barnett et al. 2013; Castle et al. 2006; Steckel et al. 2012; Tan et al. 2006).

In some of the main cotton-producing regions in the United States, *pat*-containing insect-resistant cotton cultivars have been widely used and have exhibited flexibility upon the application of glufosinate ammonium as a post-emergence herbicide. Although application of this herbicide on insect-resistant cotton plants is not recommended by manufacturers or even distributors, many farmers opt to use it as a weed control tool, especially to control glyphosate-resistant biotypes of *Amaranthus palmeri* S. Wats (Barnett et al. 2013).

In Brazil, especially in Mato Grosso and Bahia states, the *pat*-containing insect-resistant cultivar has been well accepted, and the area planted with this cultivar has increased over recent crop seasons. This technology allows for better pest management in farmed areas, and glufosinate ammonium has been intensively used by farmers in weed control, similar to the management of cultivars commercialized for their resistance to glufosinate ammonium.

Although glufosinate ammonium application to *pat*-containing insect-resistant cotton cultivars caused mild injury to the plants, it did not reduce yield (Barnett et al. 2012; Culpepper et al. 2009; Steckel et al. 2012). However, there is little published information regarding the levels of expression of the *bar* and *pat* genes, PAT activity, and the associated physiological effects of glufosinate ammonium on these transgenic cultivars relative to conventional cultivars. Thus, this study aimed to understand the relationship between the physiological changes in conventional, *bar*-containing glufosinate-resistant and *pat*-containing insect-resistant cotton cultivars after the application of different glufosinate doses and the different levels of expression of the *pat* and *bar* genes and the relative activities of phosphinothricin acetyltransferases.

## Materials and methods

### Plant growth and glufosinate application

Two greenhouse experiments were conducted involving the same treatments but with different assessments. Cotton plants of the cultivars FM 993 (non-transgenic, FiberMax, Bayer CropScience), FM 975WS (*pat*-containing insect-resistant, WideStrike^®^, Fiber Max, Dow Agrosciences), and IMACD 6001LL (LibertyLink^®^, Mato Grosso Cotton Institute—*Instituto Mato*-*Grossense de Algodão*) were grown in plastic pots filled with substrate comprising plant-based organic matter and expanded vermiculite. The substrate was previously amended regarding fertility to allow for good plant development conditions. Two cotton plants were used per pot, and the experiments followed a completely randomized design, with four replicates.

Glufosinate ammonium (Finale^®^ 200 SL, Bayer CropScience AG, Frankfurt, Germany) was applied at two time-points at doses of 200, 400, and 600 g ai ha^−1^; control plants did not receive any herbicide. The first herbicide application was done when cotton plants had two fully expanded true leaves (25 days after emergence—DAE) and the second application was when the plants had five fully expanded true leaves (40 DAE). The dose applied to each plant was the same for both applications. The conventional cultivar did not receive a second application due to the intensity of the injuries caused by the first application.

### RNA isolation from cotton

Total RNAs were isolated from 21-day-old flash frozen cotton leaves using an RNeasy plant mini kit (Qiagen, Valencia, CA 91355) according to the manufacturer’s instructions. RNAs were then treated with RNase-free DNase I kit to remove residual DNA contamination and repurified with RNeasy MinElute Cleanup Kit (Qiagen, Valencia, CA 91355) according to the manufacturer’s procedures. RNA recovery and purity were determined spectrophotometrically using a NanoDrop device (ND-1000; Thermo Scientific, West Palm Beach, FL 33407) for these samples, and sample integrity was also assessed by agarose gel electrophoresis. The quality and quantity of prepared total RNA were accessed according to the MIQE Guidelines (Bustin et al. 2009, 2010).

### Quantitative real time RT-PCR (RT-qPCR) analysis

RT-qPCR was performed in triplicate using CFX96 Touch™ Real-Time PCR Detection System (Bio-Rad, Hercules, California 94547). First strand cDNA was synthesized using iScript Advanced cDNA Synthesis Kit for RT-qPCR (Bio-Rad, Hercules, California 94547) in a 20 μL reaction with 1 µg of total RNA as template, and then diluted into 2 ng μL^−1^ with PCR grade water (Sigma-Aldrich, St Louis, MO 63103) for PCRs. The qPCRs were conducted in a final volume of 20 μL containing 5 μL of diluted first strand cDNA, 5 pmol of each forward and reverse primer, 10 µL iTaq SYBR Green Supermix (Bio-Rad, Hercules, California 94547) with conditions of 95 °C for 30 s, 40 cycles of 95 °C for 5 s, 60 °C for 30 s, and then increasing the temperature by 0.5 °C every 5 s to access the product melt curve according to the recommendations of the manufacturer. Primers with melting temperature of 60 °C were designed using Primer3 program (Koressaar and Remm 2007; Untergasser et al. 2012) under its default settings. The primers used for each gene are provided in Table 1. Primer efficiency curves were conducted using a tenfold serial dilution of cDNA samples, ranging from 0.0001 to 100 ng (equivalent of 0.0001–100 ng total RNA). Primer efficiency and slope were 97.2 % and −3.444 (*R*^2^ = 0.997) for *bar* gene, 105.5 % and −3.207 (*R*^2^ = 0.992) for *pat*, 99.2 % and −3.341 (*R*^2^ = 0.998) for UBQ14, 98.2 % and −3.365 (*R*^2^ = 0.996) for GAPDH, and 100.8 % and –3.303 (*R*^2^ = 0.992) for PP2A. The relative expression level of *bar* gene and *pat* gene was calculated using Bio-Rad CFX Manager software (version 3.1). All values were normalized to the expression values of three reference genes (UBQ14, PP2A, and GAPDH (Artico et al. 2010; Wang et al. 2013).

**Table 1:** Primer sequences for TR-qPCR

Gene	Oligo name	Sequence (reads 5′–3′)
*Pat*	patF	ACGATCCATCTGTTAGGTTGCA
	patR	CCATCCACCATGCTTGTATCCA
*Bar*	barF	GCTCTACACCCACCTGCTG
	barR	CAGCCCGATGACAGCGAC
*UBQ14*	UBQ14F	CAACGCTCCATCTTGTCCTT
	UBQ14R	TGATCGTCTTTCCCGTAAGC
*PP2A*	PP2A1F	CACTGCCCTGATTGAAAGTCAG
	PP2A1R	GTCCAGAGCACGGATGTTATCT
*GAPDH*	GAPDHF	TGATGCCAAGGCTGGAATTGCTT
	GAPDHR	GTGTCGGATCAAGTCGATAACACGG

*GAPDH* glyceraldehyde-3-phosphate dehydrogenase C subunit, *PP2A* protein phosphatase 2A, *UBQ14* polyubiquitin

### Preparation of total soluble protein extract

Cotton leaf material was collected from seedlings grown in a growth chamber to their second true-leaf stage, flash frozen in liquid nitrogen, and stored in −80 °C freezer. Extraction of PAT from plant samples was modified from a previous method (Dröge et al. 1992). Three grams of frozen leaf was ground in a mortar and pestle and collected in 2.5 mL of extraction buffer (0.5 M Tris–HCl, 0.4 mM EDTA; 2 mM dithiothreitol, and 0.3 mg mL^−1^ bovine serum albumin, pH 7.5 on ice). The extract was centrifuged in for 15 min at 16,000×*g* and 4 °C in a refrigerated microcentrifuge (Sorvall Fresco, Thermo Scientific, West Palm Beach, FL 33407). The supernatant was collected (3 mL) and 30 μL of protease inhibitor cocktail for plant cell and tissue extracts (P9599, Sigma-Aldrich, St. Louis, MO, 63103) was added. The sample was centrifuged as described above for 5 min. The supernatant was collected and loaded on a PD10 column pre-equilibrated with assay buffer (50 mM Tris–HCl, 0.4 mM EDTA, 2 mM DTT, and 0.3 mg mL^−1^ bovine serum albumin, pH 7.5 at 37 °C). The amount of total soluble protein was determined using the Bradford assay (Bradford 1976).

### PAT enzyme assay in cotton crude extracts

Assay for PAT activity was modified from a previous protocol (Wehrmann et al. 1996). [^14^C]Acetyl-CoA with a specific activity of 55 mCi mmol^−1^ was purchased from American Radiochemicals Inc. (St. Louis, MO 63146). Each assay consisted of a 40 μL aliquot of extract incubated with 5 μL of d,l-phosphinothricin (PPT, glufosinate from ChemService, West Chester, PA 19381) (from 50 mM stock) and 5 μL of [^14^C]acetyl-CoA (10 mM with 400,000 dpm) for 5, 15, 30, and 60 min at 30 °C. The reaction was stopped by adding 1 mL of 5 % NH_4_OH in water (v/v). The acetylated PPT was trapped in a strong anion solid phase column (Oasis MAX 500 mg LP extraction cartridges, Waters, Milford, MA, USA) as follows.

The solid phase column was first washed with 15 mL of ACN and then 15 mL H_2_O. The 1 mL stopped reaction was loaded on the column and washed with 9 mL of 5 % NH_4_OH in water (v/v), 10 mL of ACN, and 10 mL of 3 % acid in ACN (v/v). N-acetylated PPT was eluted with 5 mL of 5 % acid in water (v/v), mixed with 15 mL of Ultima Gold scintillation fluid (Packard BioScience, Meriden, CT). The amount of radioactivity was quantified with a Packard TriCarb 1600R Scintillation Counter (Perkin Elmer, Waltham, MA).

### Assessment of the plant injury and electron transport rate (ETR)

Plant injury was visually assessed at 2, 3, 5, 8, 15, 22, 29, and 36 days after treatment (DAT) by assigning scores between 0 and 100 (0 corresponds to the absence of symptoms and 100 to plant death).

The ETR in photosystem II was assessed immediately before herbicide application and at 0, 1, 2, 5, and 8 DAT for the FM 993 cultivar and immediately before herbicide application and at 1, 2, 5, 8, 15, 17, and 22 DAT for the FM 975WS and IMACD 6001LL cultivars. Eight replicates of the ETR readings were performed per treatment using a portable fluorometer (Multi-Mode Chlorophyll Fluorometer OS 5p—Opti Sciences, Hudson, NH 03051). A light-emitting diode (LED) source with a red light peak at the 660 nm wavelength was used, for which radiation higher than 690 nm was blocked. The mean light intensity was adjusted to between 0 and 1 μmol m^−2^ s^−1^ using a 35 W halogen lamp. The beam was optically monitored inside the chamber to correct for variations due to the changes in ambient temperature, and the optical signals were transferred to the leaf surface by an optical fiber, obtaining a 2-cm^2^ illuminated area. The analyses were performed following the method of Genty et al. (1989), assessing the chlorophyll fluorescence emission on the upper surface of the leaves.

### Sample collections and ammonia and glutamate extraction and quantification

Leaves were collected from all the plants at two DAT application, for both application time-points and for the different treatments tested. A portion of the plant material collected from each of the samples was stored in an ultra-freezer (−80 °C) for subsequent glutamate extraction and quantification.

Ammonia was extracted from fresh leaf tissue (5 g), immediately after collection. The samples were placed in beakers containing 300 mL of water acidified with hydrochloric acid (pH 3.5) and placed in an ultrasonic bath for 30 min. The ammonia content of the solution was determined by spectrophotometry according to published methods (Dayan et al. 2015; Wendler et al. 1990) using a spectrophotometer (Cintra 40, GBC Scientific Equipment Ltd.).

For glutamate extraction, the samples were ground in a mortar with liquid nitrogen, and then, 10 mL of a methanol:water solution (75:25) was added to 200 mg of each ground sample. This step was followed by a 30-min incubation in an ultrasonic bath and centrifugation at 4000*g* for 10 min (Barberis 2012).

The glutamate concentration was quantified in the samples by liquid chromatography and mass spectrometry (LC–MS/MS) using a high-efficiency liquid chromatography apparatus (Proeminence UFLC, Shimadzu Corporation, Kyoto, Japan) coupled to a hybrid triple quadrupole mass spectrometer (3200 Q TRAP, Applied Biosystems, Foster City, CA, USA). A Synergi 2.5 μm Fusion CY 100 Å chromatographic column was used, with 5 mM ammonium acetate in water (phase A) and 5 mM ammonium acetate in 75 % methanol (phase B) as mobile phases and with a flow rate of 0.5 mL min^−1^. The following gradient was used: 0 min, 50 % phase B; 1 min, 95 % phase B; and 6 min, 50 % phase B. The total run time was 8 min, and the retention time of the compound in the chromatographic column was 1.29 min. Positive ion mode electrospray ionization (ESI) was used.

### Data analysis

The data were subjected to analysis of variance, and the means were compared by a *t* test at 5 % probability using SAS (version 9.2; SAS Institute Inc., Cary, NC). The ETR results were transformed into a percentage ETR using the control treatment as a reference (100 %) for each cultivar. The standard errors of each mean were established (the mean ± standard error) for all the parameters assessed.

## Results

**Fig. 1:**
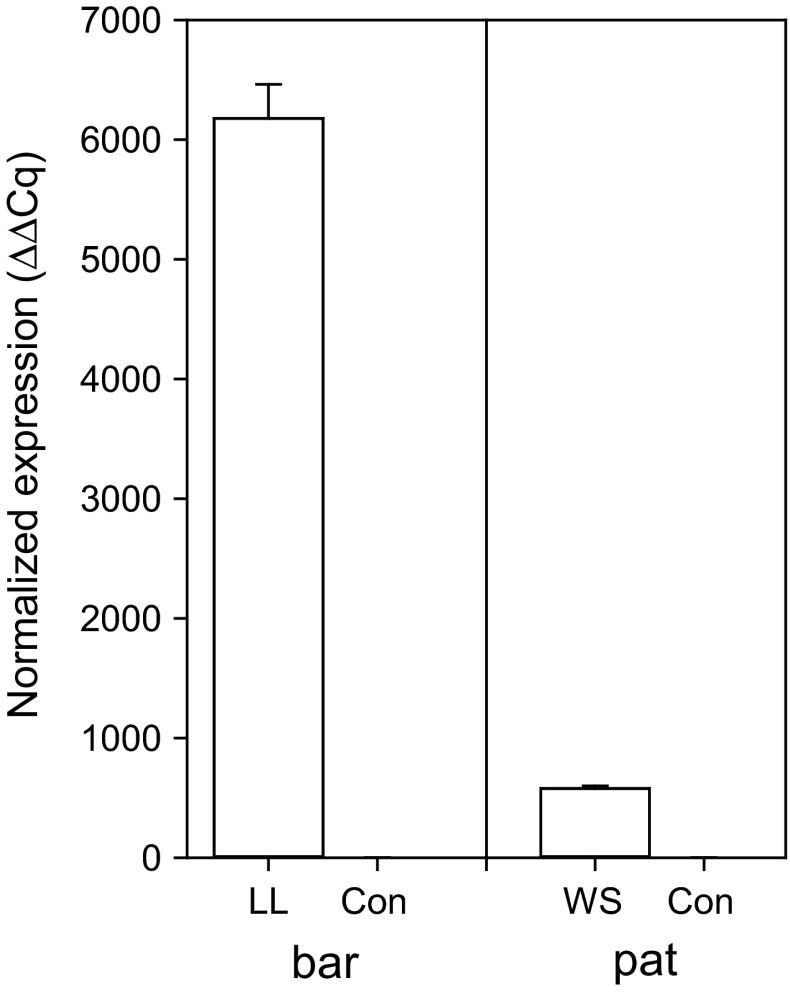
Normalized expression of the *bar* and *pat* genes in 3 weeks old leaves of conventional FM 993 versus glufosinate-resistant IMACD 6001LL, or conventional FM993 versus insect-resistant FM 975WS cotton cultivars

Relative to the expression values of three reference genes (UBQ14, PP2A, and GAPDH), young leaves of glufosinate-resistant IMACD 6001LL cotton had very high levels of *bar* gene (>6000 ΔΔCq), whereas leaves of insect-resistant FM 975WS cotton had much lower levels of the *pat* gene (ca. 500 ΔΔCq) (Fig. 1). Neither of the phosphinothricin acetyltransferase genes (*bar* or *pat*) could be detected in conventional cotton.

**Fig. 2:**
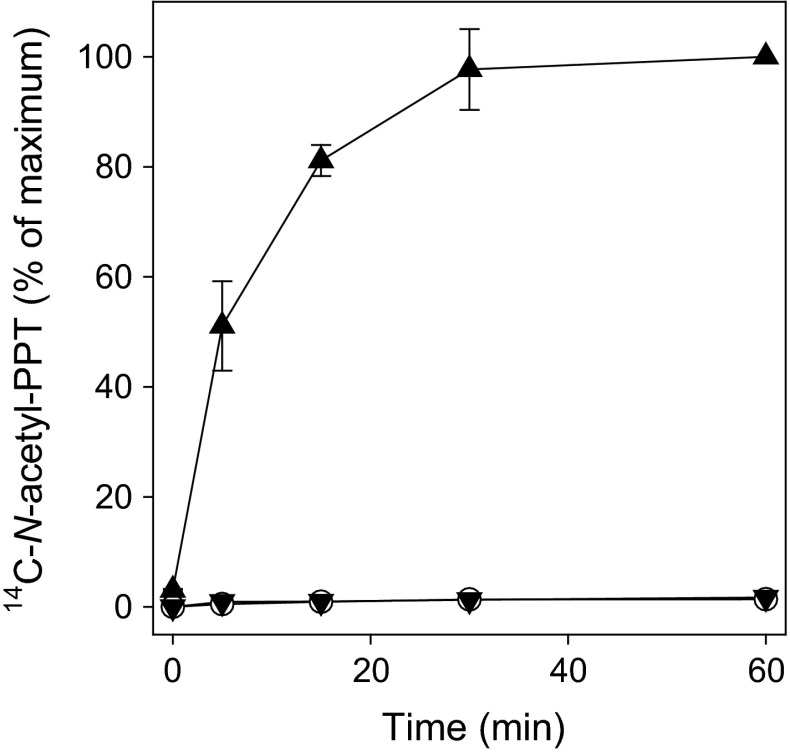
Metabolism of glufosinate by phosphinothricin acetyltransferase in cell free extracts of conventional FM 993 (*circle*), insect-resistant FM 975WS (*filled inverted triangle*), and glufosinate-resistant IMACD 6001LL (*filled triangle*) cotton cultivars

The high level of *bar* expression in glufosinate-resistant IMACD 6001LL was commensurate with very high level of PAT activity in cell free extracts. Under the conditions of the in vitro assay, 100 % of glufosinate (250 nmol) was acetylated within the first 60 min of incubation (Fig. 2). Although low levels of the *pat* gene were measured in insect-resistant FM 975WS, no PAT activity was detected in the enzyme assay. On the other hand, the lack of PAT activity in conventional cotton is consistent with the fact that it does not have either of the phosphinothricin acetyltransferase genes.

**Fig. 3:**
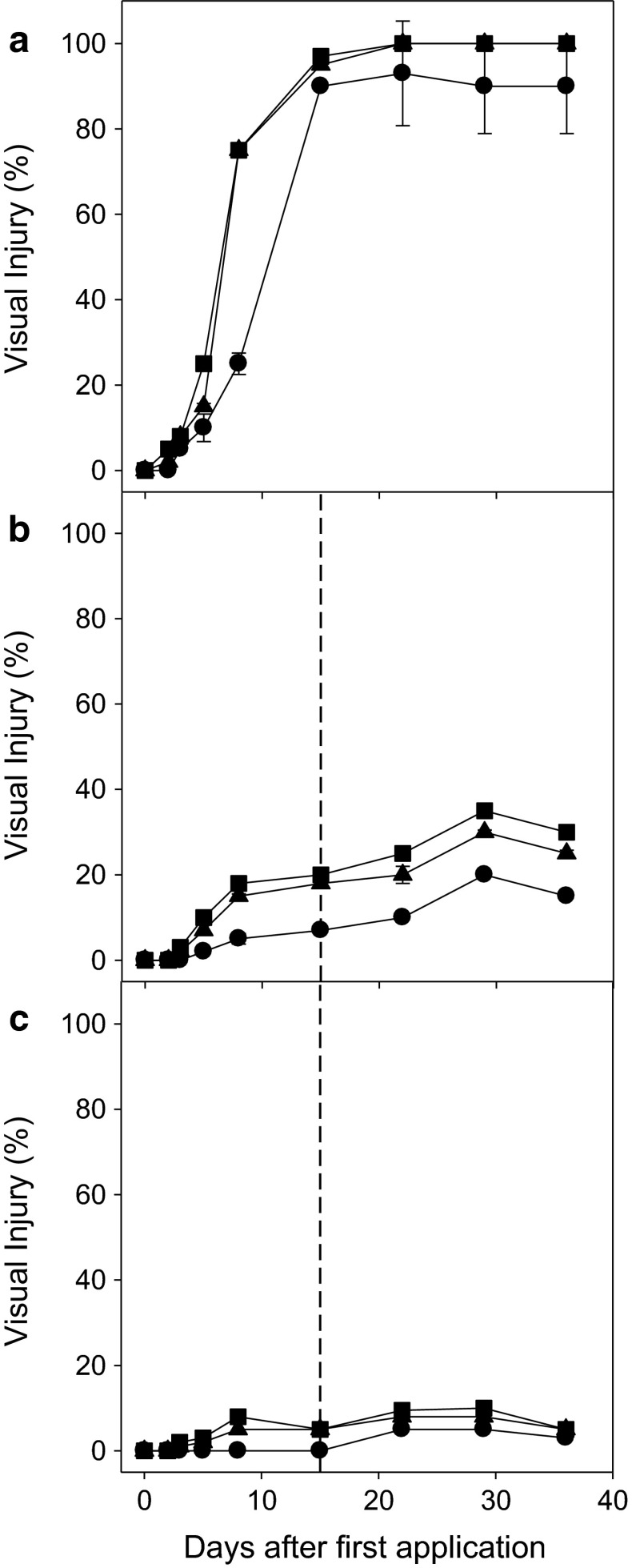
Percentage of visual injury in, **a** conventional FM 993, **b** glufosinate-resistant IMACD 6001LL, and **c** insect-resistant FM 975WS cultivars after the application of 200 (*filled* *circle*), 400 (*filled triangle*), and 600 (*filled square*) g ai ha^−1^ glufosinate ammonium (the *dashed line* represents the moment of the second application). Note: Y axis is note in the same scale for the different cultivars

Since conventional cotton is not intrinsically resistant to glufosinate, these plants were severely affected by all the glufosinate doses tested. The highest levels of injury ranged from 90 to 100 at 15 DAT in plants treated with 400 and 600 g ha^−1^ (Fig. 3). Plants were not treated a second time because most of the samples were too damaged by the first treatment.

For the glufosinate-resistant cultivar (IMACD 6001LL), the levels of injury were very low regardless of the glufosinate dose and number of applications. The highest injury percentages were observed for the highest dose after the second application, which demonstrates the high level of resistance conferred by the *bar* gene.

**Fig. 4:**
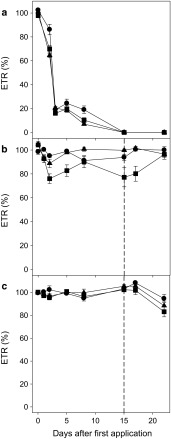
Electron transport rate (ETR) in photosystem II in **a** conventional FM 993, **b** glufosinate-resistant IMACD 6001LL, and **c** insect-resistant FM 975WS cultivars after the application of 200 (*filled* *circle*), 400 (*filled triangle*), and 600 (*filled square*) g ai ha^−1^ glufosinate ammonium (the *dashed line* represents the moment of the second application)

Despite the presence of the *pat* gene that confers glufosinate resistance, the insect-resistant cultivar is not commercialized as an herbicide-resistant transgenic crop. It exhibited higher levels of injury than the commercial glufosinate-resistant cultivar. The effects were proportional to the doses applied; however, the level of injury was much lower than that of the conventional cultivar (Fig. 4).

Glufosinate had a similar effect on photosynthetic ETR as observed with injury, with intermediate sensitivity for the insect-resistant cultivar relative to the responses of the glufosinate-sensitive conventional and glufosinate-resistant cultivars (Fig. 4). On the second DAT, the conventional cultivar (FM 993) exhibited a pronounced decline in ETR at all the doses applied. This decline was proportional to the dose tested, although the differences between doses were small.

The ETR values in the resistant cultivar remained unaltered by the first glufosinate application at any dose tested. After the second application, there was a small decline in ETR at the 400 g ha^−1^ dose, which was more pronounced at the 600 g ha^−1^ dose. However, this decline was already much lower than the decrease observed for the conventional cultivar after the first application (Fig. 4). Nevertheless, after the second application at the highest dose tested, there was enough glufosinate to cause a small reduction in photosynthesis (Fig. 4).

The insect-resistant FM 975WS cultivar exhibited a small decline in ETR starting with the first application at the higher glufosinate doses tested. However, the plants exhibited recovery of the ETR, which decreased again starting from the second application at the same dose and increased again at seven DAT.

Regarding ammonia and glutamate levels (Tables 2, 3, respectively), which are both substrates of the reaction catalyzed by GS, the levels of these compounds are naturally different in the different cultivars without glufosinate application. Levels of these metabolites increased in the conventional cultivar, especially ammonia, after glufosinate application, and these increases were directly proportional to the dose applied. A second application was not performed for this cultivar due to the high intensity of plant injury or death caused by the first application.

**Table 2:** Ammonia content (mg ammonia kg^−1^ fresh weight) in different cotton cultivar plants after glufosinate application

Glufosinate (g ai ha^−1^)	First application	Second application
Conventional cotton (FM 993)		
0	16.92 ± 0.64 a	–
200	68.99 ± 12.16 bc	–
400	79.34 ± 6.75 bc	–
600	166.35 ± 21.85 c	–
Glufosinate-resistant cotton (IMACD 6001LL)		
0	54.27 ± 7.13 a	57.3 ± 5.69 a
200	38.67 ± 4.73 a	62.15 ± 7.42 a
400	47.06 ± 6.51 a	56.41 ± 6.04 a
600	51.26 ± 18.14 a	64.02 ± 10.02 a
Insect-resistant cotton (FM 975WS)		
0	44.30 ± 4.54 a	78.48 ± 11.32 a
200	16.13 ± 6.00 a	54.09 ± 6.08 a
400	44.76 ± 12.91 a	85.14 ± 17.79 a
600	123.01 ± 20.79 b	134.52 ± 14.66 b

Data represent the means of 4 replications ± standard error. Means followed by the same letter in the columns do not statistically differ from each other by the *t* test (*p* > 0.05)

**Table 3:** Glutamate content (mg glutamate kg^−1^ fresh weight) in different cotton cultivar plants after glufosinate application

Glufosinate (g ai ha^−1^)	First application	Second application
Conventional cotton (FM 993)		
0	2.64 ± 0.23 a	–
200	7.11 ± 2.68 a	–
400	18.41 ± 2.38 b	–
600	27.01 ± 2.87 c	–
Glufosinate-resistant cotton (IMACD 6001LL)		
0	2.56 ± 0.48 a	18.4 ± 5.00 ab
200	2.22 ± 0.18 a	15.14 ± 4.71 a
400	3.81 ± 0.58 a	31.03 ± 5.88 b
600	3.82 ± 0.84 a	56.65 ± 4.58 c
Insect-resistant cotton (FM 975WS)		
0	7.16 ± 2.38 a	2.85 ± 0.55 a
200	7.70 ± 3.70 a	12.77 ± 4.03 ab
400	5.24 ± 0.74 a	18.09 ± 6.13 b
600	6.21 ± 1.11 a	14.53 ± 4.28 ab

Data represent the means of 4 replications ± standard error. Means followed by the same letter in the columns do not statistically differ from each other by the *t* test (*p* > 0.05)

Although there was a small non-significant difference in ammonia levels for the 200 and 400 g ha^−1^ doses, plants only died at the 400 g ha^−1^ dose (Table 2). The insect-resistant FM 975WS cultivar did not have increased ammonia levels for the 200 and 400 g ha^−1^ doses, even after the second application. Only the highest glufosinate dose caused significantly increased ammonia levels after two applications, suggesting that this cultivar has a lower level of resistance than the resistant cultivar, but it is still quite satisfactory because it was not significantly affected at the two lowest doses tested. The presence of the resistance gene, used as a marker, ensures a good level of resistance to glufosinate, though it is lower than the level of resistance of the cultivar exclusively transformed for this purpose.

There was a small change in the glutamate levels in the resistant cultivar at the two highest glufosinate doses after the first application and more marked changes after the second application at the same doses. The same phenomenon occurred for the insect-resistant FM 975WS cultivar starting, however, from the lowest dose after the second application.

## Discussion

Glufosinate resistance in IMACD 6001LL cotton is achieved by the insertion of the *bar* gene derived from *S. hygroscopicus*, strain ATCC 21705. This bacterial gene was codon-optimized for improved translation in plants. Furthermore, the gene was placed under the control of a CaMV 35S constitutive promoter and the construct includes a 3′-nos sequence (nopaline synthase gene from the pTiT37 plasmid of *A. tumefaciens*) as a terminator element. This construct insures high level of *bar* expression and high resistance to glufosinate (Hérouet et al. 2005). On the other hand, the insect-resistant FM 975WS cotton is a transgenic plant that has two Cry genes (Cry1A and Cry1F) that confer resistance to pests (Castle et al. 2006). In these cultivars, the *pat* gene from *S. viridochromogenes* is used as a selectable marker gene coexpressed in association with the Cry1Ac and Cry1F genes under either (4OCS)Δmas2′ (mannopine synthase promoter including four copies of the ocs enhancer element of the octopine synthase gene from *Agrobacterium tumefaciens*) or uBiZM1 (ubiquitin from *Zea mays*) constitutive promoters. Both use the terminator element ORF25PolyA. While the constructs inserted in cotton provides some level of tolerance to glufosinate, it is not meant to impart resistance to field rates of the herbicide (OECD 2002; Tan et al. 2006). Furthermore, it has been reported that subcellular localization rather than the absolute amount of the enzyme is critical for direct selection of transgenic clones (Lutz et al. 2001). Accordingly, the level of expression of *pat* and overall PAT enzyme activity in insect-resistant FM 975WS variety used in this study were much lower than that of *bar* in glufosinate-resistant IMACD 6001LL (Figs. 1, 2).

One of the secondary effects of the phytotoxic response to glufosinate is reduced photosynthetic ETRs. This has been observed in white mustard (Ziegler and Wild 1989), soybean (Barberis 2012) and cucumber plants (Dayan and Zaccaro 2012) treated with glufosinate. The extremely rapid acetylation of l-phosphinothricin into non-toxic *N*-acetyl-l-phosphinothricin metabolite achieved in glufosinate-resistant IMACD 6001LL (Fig. 2) (Dröge et al. 1992; OECD 2002) protected this cultivar from inhibition of rubisco enzyme activity and overall photosynthetic activity (Fig. 4).

The insect-resistant FM 975WS is considered a glufosinate-susceptible cultivar and the plants do not have fully developed detoxification mechanism against the herbicide and typically accumulate ammonia following herbicide application (Manderscheid et al. 2005; Wild et al. 1987).

Application of glufosinate causes ammonia accumulation in most plant species, including *Sinapis alba* (Wild et al. 1987), *Sinapis alba* and maize (Wendler et al. 1990), *Brassica napus* (Downs et al. 1994), *Setaria viridis* and barley (Mersey et al. 1990), *Amaranthus palmeri* (Coetzer and Al-Khatib 2001), *Abutilon theophrasti* (Sellers et al. 2004), *Chenopodium album*, *Solanum nigrum*, *Tripleurospermum inodorum* and *Echinochloa crus*-*galli* (Manderscheid et al. 2005), rice (Tsai et al. 2006), and soybean (Pornprom et al. 2000).

Ammonia levels did not increase in the glufosinate-resistant cotton cultivar plants regardless of the dose tested, even with the second application at the highest dose (Table 2). The lack of ammonia accumulation indicates that GS activity was not impaired, most likely due to the rapid metabolism of the herbicide by PAT (Fig. 2) (Manderscheid and Wild 1986).

Glufosinate is a structural analogue of glutamate that binds irreversibly to GS and inhibits glutamine synthesis (Gill and Eisenberg 2001; Manderscheid and Wild 1986). This can lead to an increase in glutamate content, as was observed in some of our experiments and reported by others before (Barberis 2012). Overall, the quantification of the two substrates of GS (ammonia and glutamate), the ETR in photosystem II, and the level of plant injury indicated that the insect-resistant cultivar had a good level of resistance to glufosinate ammonium. Consistent with other reports (Sweeney and Jones 2015), the level of resistance to this herbicide measured herein was slightly lower than that of the herbicide-resistant IMACD 6001LL cotton cultivar (Fig. 3).

While Dow Agrosciences does not encourage the use of glufosinate in a post-emergence broadcast setting on the insect-resistant FM 975WS cotton because it may cause up to 25 % crop injury (Stewart et al. 2013), the US Environmental Protection Agency has approved the use of this herbicide on these cotton varieties. The crop safety margin is dependent on the plant development stage at application and the doses used for that cultivar (Wright et al. 2014), with higher injury and reduced yield resulting from late application compared to early application (Barnett et al. 2013; Sweeney and Jones 2015). However, the fiber yield is often not affected or even improved, and the use of these varieties for their herbicide resistance trait in addition to their resistance to insects is widespread in the southern US (Culpepper et al. 2009; Stewart et al. 2013; Whitaker et al. 2011).

### *Author contribution statement*

CAC, DOL, GLGCG and EDV conceived, designed and conducted field and physiological research. DKO, ZP and FED conceived, designed, and conducted the biochemical and molecular biology research. All authors contributed to data analysis and writing the manuscript
